# Synthesis and Antifungal Activity of Carabrone Derivatives

**DOI:** 10.3390/molecules15096485

**Published:** 2010-09-16

**Authors:** Jun-Tao Feng, Zhi-Qing Ma, Jiang-Hua Li, Jun He, Hui Xu, Xing Zhang

**Affiliations:** 1 Research and Development Center of Biorational Pesticide, College of Plant Protection, Northwest A&F University, Yangling 712100, China; 2 Laboratory of Pharmaceutical Design & Synthesis, College of Sciences, Northwest A&F University,Yangling 712100, China

**Keywords:** carabrone, structural modification, synthesis, antifungal activity

## Abstract

Nine derivatives **6-14** of carabrone (**1**) were synthesized and tested *in vitro* against *Colletotrichum lagenarium* Ell et Halst using the spore germination method. Among all of the derivatives, compounds **6-8** and **12** showed more potent antifungal activity than **1**. Structure-activity relationships (SAR) demonstrated that the* γ*-lactone was necessary for the antifungal activity of **1**, and the substituents on the C-4 position of **1** could significantly affect the antifungal activity.

## 1. Introduction

**Figure 1:**
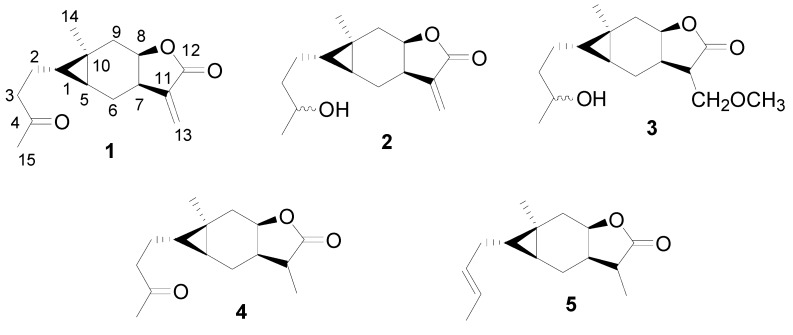
The chemical structures of carabrone and its derivatives.

Carabrone (**1**, Figure 1), containing cyclopropane and sesquiterpene lactone moieties, was first isolated from the fruits of *Carpesium abrotanoides* [1], and is widely distributed in feverfew and other plant species [2,3,4,5,6,7,8,9]. It was demonstrated that compound **1** displays cytotoxic [10], antibacterial [11,12], and antitumor activity [13]. In our course of screening for novel naturally occurring phytofungicides from the plants in northwestern China, compound **1 **was obtained from *Carpesium macrocephalum*, and exhibited antifungal activities *in vitro* and *in vivo* against *Botrytis cinerea*, *Colletotrichum lagenarium*, and *Erysiphe graminis* [14]. Subsequently, we prepared four derivatives (**2-5**,**Figure 1**) from **1**, and found that the 11,13-double bond and the carbonyl group on the C-4 position of **1 **are two active sites [15,16]. In order to further investigate the effect of lactone and substituents on the C-4 position of **1** on the antifungal activity, herein we synthesized nine new carabrone derivatives **6**-**14** as potential antifungal agents.

## 2. Results and Discussion

**Scheme 1:**
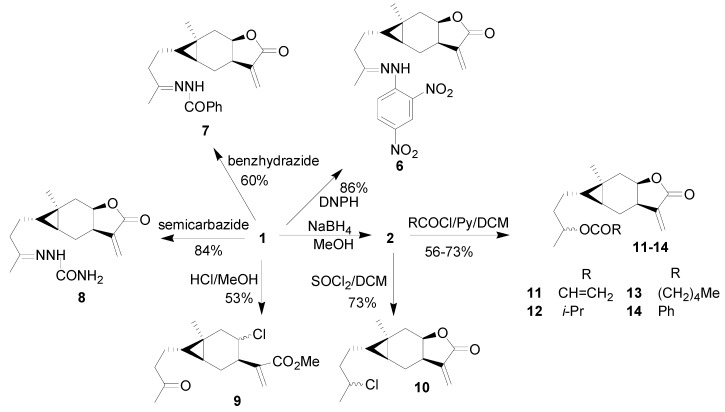
The synthetic route to carabrone derivatives **6-14**.

Nine carabrone derivatives **6**-**14** were synthesized as shown in Scheme 1. Compound **6** was prepared by the reaction of 2,4-dinitrophenyl hydrazine (DNPH) and **1** in the presence of hydrogen chloride (HCl). Benzhydrazide or semicarbazide reacted with **1** to give compounds **7** and **8**, respectively. Compound **9** was prepared from **1 **with dry HCl. Compound **10** was synthesized by the reduction of the carbonyl group of **1** in the presence of NaBH_4_, followed by chlorination of the 4-OH group of **2** with thionyl chloride (SOCl_2_). Compound **2** reacted with acyl chlorides in the presence of pyridine to afford compounds **11**-**14**. All compounds were characterized by ­^1^H-NMR, IR, and HR-MS spectra. 

The antifungal activity was assayed *in vitro* against *Colletotrichum lagenarium* Ell *et* Halst by the spore germination method. Chlorothalonil was used as a positive control. As described in Table 1, compounds **6**-**8** exhibited the most potent antifungal activity with the EC_50_ values of 2.24, 4.32 and 3.03 μg/mL, respectively, *i.e*., the antifungal activity of **6**, **7** and **8** was 1.5-3 times more potent than that of **1**. However, the antifungal activity of other compounds was 1-8 times less than that of **1**. Obviously, substituents on the C-4 position of **1** could significantly affect the antifungal activity. For example, introducing the hydrazone substituents on the C-4 position of **1 **lead to the most potent compounds (e.g., **6**-**8**), while when other substituents, such as the hydroxy group, chloro atom, and ester groups (except isobutyryloxy group), were introduced on the C-4 position of **1**, the corresponding compounds showed the less potent activity than **1 **(e.g., **10**, **11**, **13** and **14**). Interestingly, when the isobutyryloxy group was introduced on the C-4 position of **1** to give **12**, the EC_50_ value of **12** was 6.39 μg/mL, which was more potent than that of **1**. Meanwhile, compound **1** was nearly eightfold more potent than **9** (EC_50_ 7.10 μg/mL for **1 ***vs.* EC_50_ 56.30 μg/mL for **9**). This demonstrated that the* γ*-lactone was necessary for the antifungal activity of **1**, and opening the lactone would lead to a less potent compound (**1** *vs. ***9**).

**Table 1:** Inhibition rates of carabrone derivates (**6-14**) against spore germination of *Colletotrichum lagenarium.^a^*

Compd.	Regression equation (Y = a + bX)	r	EC_50_*^b^* (μg/mL)	EC_50 _95% *CL*/(μg/mL)
**1**	Y = 3.6090 + 1.6337X	0.9974	7.10	6.19～8.02
**6**	Y = 4.5130 + 1.3891X	0.9923	2.24	1.97～2.55
**7**	Y = 3.4038 + 2.5118X	0.9817	4.32	3.81～4.85
**8**	Y = 4.3577 + 1.3351X	0.9979	3.03	2.58～3.55
**9**	Y = 3.1867 + 1.0358X	0.9942	56.30	42.95～73.80
**10**	Y = 3.2442 + 1.1736X	0.9969	31.34	26.08～37.66
**11**	Y = 4.2780 + 0.6720X	0.9882	10.78	9.16～12.68
**12**	Y = 4.3568 + 0.7209X	0.9920	6.39	5.33～7.65
**13**	Y = 3.7775 + 0.9365X	0.9970	20.20	16.85～24.22
**14**	Y = 3.7676 + 1.0011X	0.9962	17.02	14.41～20.11
chlorothalonil *^c^*	Y = 5.1247 + 1.0081X	0.9935	0.75	0.63～0.90

***^a ^*** Values are means of three separate experiments; *^b^* EC_50 _(50% effective concentration), concentration of compound that reduces spore germination by 50%; *^c ^*Chlorothalonil was used as a positive control.

## 3. Experimental

### 3.1. General

All the solvents were of analytical grade and the reagents were commercially available. Thin-layer chromatography (TLC) and silica gel-column chromatography were performed with silica gel plates using silica gel 60 GF­­_254_, and 200-300 mesh (Qingdao Haiyang Chemical Co., Ltd., China). Melting points were determined on a digital melting-point apparatus and uncorrected. All compounds were characterized by proton nuclear magnetic resonance (^1^H-NMR), high-resolution mass spectra (HR-MS), mass spectra (MS-ESI), and infrared spectra (IR), respectively. 

### 3.2. Synthesis

*(3aR, 4aS, 5S, 5aR, 6aR)-5-(3-keto 2,4-dinitrophenyl hydrazone-butyl)-5a-methyl-3-methylene-3a, 4, 4a, 5, 6, 6a-hexahydrocyclopropa[f]benzofuran-2-one*** (6)**. A mixture of compound **1 **(124 mg, 0.5 mmol), 2,4-dinitrophenyl hydrazine (DNPH, 39.6 mg, 2 mmol) and hydrochloric acid (0.2 mL, 6 mol/L) in anhydrous methanol (MeOH, 10 mL) was reacted at 60 ºC until a precipitate formed. The reaction mixture was then filtered, and the filtrate was evaporated under educed pressure. The residue was recrystallized in dimethyl sulfoxide (DMSO) to produce **6** as a yellow solid. Yield: 86%, m.p. 181–182 ºC [17]; IR (KBr) cm^-1^: 3321, 2956, 1756, 1593, 1530, 1345; ^1^H-NMR (400 MHz, CDCl_3_) *δ*: 10.79 (s, 1H, =NNH), 8.84 (m, 1H, H-3´), 8.35 (d, *J* = 6.8 Hz, 1H, H-5´), 7.82 (d, *J* = 10.0 Hz, 1H, H-6´), 5.99 (d, *J* = 2.4 Hz, 1H, H-13), 5.62 (d, *J* = 2.4 Hz, 1H, H-13), 4.80 (m, 1H, H-8), 3.31 (m, 2H), 3.18 (m, 1H, H-7), 2.43~2.55 (m, 1H), 2.26~2.35 (m, 1H), 2.05 (s, 3H, H-15), 1.54~1.64 (m, 2H), 1.05 (s, 3H, H-14), 0.85~0.91 (m, 2H), 0.53 (m, 1H, H-5), 0.35 (m, 1H, H-1); HR-MS (ESI): *m/z *calcd for C_21_H_25_N_4_O_6 _([M+H]­^+^), 429.1769; found, 429.1763.

*(3aR, 4aS, 5S, 5aR, 6aR)-5-(3-keto benzoyl hydrazone-butyl)-5a-methyl-3-methylene-3a, 4, 4a, 5, 6, 6a-hexahydrocyclopropa[f]benzofuran-2-one* **(7)**. A mixture of **1 **(125 mg, 0.5 mmol), benzhydrazide (82 mg, 0.6 mmol), and 1-2 drops of HOAc in absolute ethanol (10 mL) was stirred at 80 ºC. After 3 h, the solvent was removed under reduced pressure to give a residue, which was dissolved in CH_2_Cl_2_. Then the organic phase was washed with H_2_O, dried by anhydrous Na_2_SO_4_, and evaporated under reduced pressure. Finally, the residue was purified by silica gel-column chromatography using CH_2_Cl_2_-EtOAc as the eluent to give **7** as a pale yellow solid. Yield: 60%, m.p. 45–46 ºC; ^1^H-NMR (500 MHz, CDCl_3_) *δ*: 8.77 (s, 1H, CONH), 7.79 (s, 2H, Ar-H), 7.46 (s, 1H, Ar-H), 7.42 (s, 2H, Ar-H), 6.19-6.20 (m, 1H, H-13), 5.22 (d, *J* = 2.2 Hz, 1H, H-13), 4.72-4.4.77 (m, 1H, H-8), 3.11-3.13 (m, 1H, H-7), 2.48-2.51 (m, 2H), 2.26-2.33 (m, 3H), 2.21 (s, 1H), 1.94 (s, 2H), 1.51-1.73 (m, 2H), 1.23 (s, 1H), 1.06 (s, 3H, H-14), 0.83-0.97 (m, 2H, H-2). MS (ESI): *m/z* 389 ([M+Na]­^+^), 100).

*(3aR, 4aS, 5S, 5aR, 6aR)-5-(3-keto *carbamoyl **hydrazone*-butyl)-5a-methyl-3-methylene-3a, 4, 4a, 5, 6, 6a-hexahydrocyclopropa[f]benzofuran-2-one* **(8)**. A mixture of **1 **(223.2 mg, 0.9 mmol), semicarbazide hydrochloride (111.5 mg, 1 mmol) and sodium acetate (176.8 mg, 1.3 mmol) in distilled H_2_O (5 mL) was stirred at 60 ºC until a white precipitate was produced, then the reaction mixture was filtered, and the filtrate was evaporated under reduced pressure. The residue was recrystallized from distilled H_2_O-MeOH to give **8** as a white solid. Yield: 84%, m.p. 171–173 ºC; IR (KBr) cm^-1^: 3317, 2963, 1753, 1690, 1620; ^1^H-NMR (400 MHz, CDCl_3_) *δ*: 6.14 (d, *J* = 2.6 Hz, 1H, H-13), 5.64 (d, *J* = 2.4 Hz, 1H, H-13), 4.88 (m, 1H, H-8), 4.78 (s, 1H, =NNH), 4.60 (s, 2H, O=CNH_2_), 3.36 (m, 2H), 3.20 (m, 1H, H-7), 2.24~2.27 (m, 1H), 1.97~2.13 (m, 1H), 1.85 (s, 3H, H-15), 1.81~1.84 (m, 1H), 1.43~1.51 (m, 1H), 1.04 (s, 3H, H-14), 0.85~0.92 (m, 2H), 0.53 (m, 1H, H-5), 0.38 (m, 1H, H-1); HR-MS (ESI): *m/z *calcd for C_16_H_24_N_3_O_3 _([M+H]­^+^), 306.1812; found, 306.1812. 

*4-oxo-7β-(2-methylformate) propene-8-chloro-carabrane* **(9)** [18]. Compound **1 **(248 mg, 1 mmol) was dissolved in anhydrous MeOH (40 mL) under reflux, and a flow of dry HCl was passed for 6 h. When the starting material was nearly complete as checked by TLC, the reaction mixture was cooled to room temperature. Sodium bicarbonate (NaHCO_3_, 20 mg) and distilled H_2_O (30 mL) were added to the above mixture, which was extracted with CH_2_Cl_2_ (50 mL × 3). The organic phases were combined, and washed with 2% aq. NaHCO_3_, and distilled H_2_O, dried over anhydrous Na_2_SO_4_, and evaporated under reduced pressure. Finally, the residue was purified by silica gel-column chromatography using petroleum ether-EtOAc as the eluent to give **9** as a colorless oily liquid. Yield: 53%, [α]_D_^18^ +49.7 (*_C_* 0.35, CHCl_3_); IR (KBr) cm^-1 ^2942, 1756, 1695, 1632; ^1^H-NMR (400 MHz, CDCl_3_) *δ*: 6.13 (d, *J* = 2.6 Hz, 1H, H-13), 5.58 (d, *J* = 2.4 Hz, 1H, H-13), 3.85 (m, 1H, H-8), 3.76 (s, 3H, OCH_3_), 3.31 (m, 2H), 2.82 (m, 1H, H-7), 2.26~2.13 (m, 2H), 1.94 (s, 3H, H-15), 1.58~1.63 (m, 2H), 1.16 (s, 3H, H-14), 0.86~0.93 (m, 2H), 0.54 (m, 1H, H-5), 0.32 (m, 1H, H-1); HR-MS (ESI): *m/z *calcd for C_16_H_27_NO_3_Cl ([M+NH_4 _]­^+^), 316.1697; found, 316.1695. 

*(3aR, 4aS, 5S, 5aR, 6aR)-5-(3-chloro-butyl)-5a-methyl-3-methylene-3a, 4, 4a, 5, 6, 6a-hexahydrocyclopropa[f]benzofuran-2-one* **(10) ** [19]. A mixture of **2** (100 mg, 0.4 mmol) and pyridine (0.1 mL) in anhydrous CH_2_Cl_2_ (15 mL) was stirred at 0 ºC. Thionyl chloride (SOCl_2_, 0.1 mL) was added dropwise. After the addition was complete, the mixture was stirred under reflux. When the starting material was nearly completely consumed, as checked by TLC, NaHCO_3_ (20 mg) and distilled H_2_O (10 mL) were added to the above mixture, which was extracted with CH_2_Cl_2_ (20 mL × 3). The organic phases were combined, washed with 0.3% HCl, saturated aq. Na_2_CO_3_ and brine, dried over anhydrous Na_2_SO_4_, and evaporated under reduced pressure. Finally, the residue was purified by silica gel-column chromatography using petroleum ether-EtOAc as the eluent to produce **10 **as colorless acicular crystals. Yield: 73%, m.p. 97–99 ºC; IR (KBr) cm^-1^: 2943, 1757, 1625, 1148, 628; ^1^H NMR (400 MHz, CDCl_3_) *δ*: 6.24 (d, *J *= 2.6 Hz, 1H, H-13), 5.56 (d, *J* = 2.4 Hz, 1H, H-13), 4.81 (m, 1H, H-8), 4.04 (m, 1H, H-4), 3.14 (m, 1H, H-7), 2.17~2.37 (m, 2H, H-3), 1.79~1.83 (m, 2H), 1.56 (d, *J* = 6.0 Hz, 3H, H-15), 1.43~1.46 (m, 2H), 1.09 (s, 3H, H-14), 0.88~0.95 (m, 2H), 0.47 (m, 1H, H-5), 0.38 (m, 1H, H-1); HR-MS (ESI): *m/z *calcd for C_15_H_25_NO_2_Cl ([M+NH_4 _]­^+^), 286.1568; found, 286.1565. 

#### General procedure for the synthesis of compounds 11-14 [20]

A mixture of **2** (100 mg, 0.4 mmol) and pyridine (0.1 mL) in anhydrous CH_2_Cl_2_ (15 mL) was stirred at 0 ºC. Acyl chloride (0.1 mL) in anhydrous CH_2_Cl_2_ (2 mL) was added dropwise. After the addition, the mixture was stirred under reflux. When the reaction was nearly complete, as checked by TLC, NaHCO_3_ (20 mg) and distilled H_2_O (10 mL) were added to the above mixture, which was extracted with CH_2_Cl_2_ (30 mL × 3). The organic phases were combined, washed by 0.3% HCl, saturated aq. Na_2_CO_3_ and brine, and evaporated under the reduced pressure. Finally, the residue was purified by silica gel column chromatography using petroleum ether-acetone as the eluent to give compounds **11-14**.

*(3aR, 4aS, 5S, 5aR, 6aR)-5-(3-vinylCarbonate-butyl)-5a-methyl-3-methylene-3a, 4, 4a, 5, 6, 6a-hexahydrocyclopropa[f]benzofuran-2-one*** (11)***.* Yield: 73%, a colorless oily liquid; [α]_D_^18^ +58.7 (*_C_* 0.47, CHCl_3_); IR (KBr) cm^-1^: 2977, 1757, 1715, 1642, 1203; ^1^H-NMR (400 MHz, CDCl_3_) *δ*: 6.40 (d, *J* = 16.8 Hz, 1H, CH=CH_2_), 6.23 (d, *J* = 2.8 Hz, 1H, H-13), 6.10 (m, 1H, CH=CH_2_), 5.82 (d, *J* =10.8 Hz, 1H, CH=CH_2_), 5.56 (d, *J* =2.4 Hz, 1H, H-13), 4.99 (m, 1H, H-8), 4.78 (m, 1H, H-4), 3.16 (m, 1H, H-7), 2.31~2.39 (m, 2H), 1.59~1.62 (m, 2H), 1.36~1.45 (m, 2H), 1.27 (d, *J* =6.0 Hz, 3H, H-15), 1.06 (s, 3H, H-14), 0.85~0.99 (m, 2H), 0.43 (m, 1H, H-5), 0.35 (m, 1H, H-1); HR-MS (ESI): *m/z *calcd for C_18_H_28_NO_4 _([M+NH_4 _]­^+^), 322.2013; found, 322.2008.

*(3aR, 4aS, 5S, 5aR, 6aR)-5-(3-isopropylCarbonate-butyl)-5a-methyl-3-methylene-3a, 4, 4a, 5, 6, 6a-hexahydrocyclopropa[f]benzofuran-2-one*** (12)***.* Yield: 72%; a colorless oily liquid; [α]_D_^18^ +36.4 (*_C_* 0.41, CHCl_3_); IR (KBr) cm^-1^: 2974, 1757, 1722, 1660, 1148; ^1^H-NMR (400 MHz, CDCl_3_) *δ*: 6.23 (d, *J* = 2.6 Hz, 1H, H-13), 5.56 (d, *J* = 2.4 Hz, 1H, H-13), 4.95 (m, 1H, H-8), 4.80 (m, 1H, H-4), 3.16 (m, 1H, H-7), 2.51 (m, 1H, CH(CH_3_)_2_), 2.30~2.38 (m, 2H), 1.59~1.61 (m, 2H), 1.35~1.44 (m, 2H), 1.23 (d, *J* = 6.0 Hz, 3H, H-15), 1.18 (d, *J* = 10.8 Hz, 3H, CHCH_3_), 1.14 (d, *J* = 11.2 Hz, 3H, CHCH_3_), 1.07 (s, 3H, H-14), 0.87~0.98 (m, 2H), 0.43 (m, 1H, H-5), 0.34 (m, 1H, H-1); HR-MS (ESI): *m/z *calcd for C_19_H_28_O_4_Na ([M+Na]­^+^), 343.1880; found, 343.1875.

*(3aR, 4aS, 5S, 5aR, 6aR)-5-(3-pentylCarbonate-butyl)-5a-methyl-3-methylene-3a, 4, 4a, 5, 6, 6a-hexahydrocyclopropa[f]benzofuran-2-one*** (13)***. *Yield: 70%; a colorless oily liquid; [α]_D_^18^ +49.4 (*_C_* 0.52, CHCl_3_); IR (KBr) cm^-1^: 2935, 1722, 1660, 1146; ^1^H-NMR (400 MHz, CDCl_3_) *δ*: 6.23 (d, *J* = 2.6 Hz, 1H, H-13), 5.56 (d, *J* = 2.4 Hz, 1H, H-13), 4.95 (m, 1H, H-8), 4.78 (m, 1H, H-4), 3.16 (m, 1H, H-7), 3.01~3.04 (m, 1H, O=CCH_2_), 2.51~2.54 (m, 1H, O=CCH_2_), 2.30~2.38 (m, 2H), 1.59~1.61 (m, 2H), 1.27~1.51 (m, 8H), 1.23 (d, *J* = 6.0 Hz, 3H, H-15), 1.06 (s, 3H, H-14), 0.90~0.99 (m, 2H), 0.86~0.89 (m, 3H, CH_2_CH_3_), 0.43 (m, 1H, H-5), 0.35 (m, 1H, H-1); HR-MS (ESI): *m/z *calcd for C_21_H_36_NO_4 _([M+NH_4_]­^+^), 366.2639; found, 366.2632.

*(3aR, 4aS, 5S, 5aR, 6aR)-5-(3-phenylCarbonate-butyl)-5a-methyl-3-methylene-3a, 4, 4a, 5, 6, 6a-hexahydrocyclopropa[f]benzofuran-2-one*** (14)***.* Yield: 56%; a colorless oily liquid; [α]_D_^18 ^+73.9 (*_C_* 0.31, CHCl_3_); IR (KBr) cm^-1^: 2942, 1757, 1711, 1485, 715; ^1^H-NMR (400 MHz, CDCl3) *δ*: 8.03 (m, 2H, H-3´, 5´), 7.57 (m, 1H, H-4´), 7.46 (m, 2H, H-2´, 6´), 6.24 (d, *J* = 2.6 Hz, 1H, H-13), 5.55 (d, *J* = 2.4 Hz, 1H, H-13), 5.18 (m, 1H, H-8), 4.77 (m, 1H, H-4), 3.15 (m, 1H, H-7), 2.28~2.36 (m, 2H), 1.63~1.79 (m, 2H), 1.42~1.56 (m, 2H), 1.23 (d, *J* = 6.0 Hz, 3H, H-15), 1.08 (s, 3H, H-14), 0.88~0.92 (m, 2H), 0.47 (m, 1H, H-5), 0.35 (m, 1H, H-1); HR-MS (ESI): *m/z* calcd for C_22_H_30_NO_4 _([M+NH_4_]­^+^), 372.2169; found, 372.2168.

### 3.3. Spore Germination Assay

Microorganisms and maintenance: the strain of *Colletotrichum lagenarium* (36199) was provided by Agricultural Culture Collection of China and maintained on potato dextrose agar (PDA). Compounds **1** and **6-14** were dissolved in acetone or DMSO and added to 2% water agar medium after sterilization to produce concentrations of 100, 75, 50, 25, 10, and 5 µg/mL or 10, 5, 2, 1, 0.5 and 0.25 µg/mL of medium. Conidial suspensions (0.2 mL) containing 1 × 10^5^ condia/mL, derived from cultures grown for 12 d on PDA plates, were spread on 2% water agar. Conidia were allowed to germinate 25 ± 1 ºC for 8 h. Germination was quantified at three sites by counting 100 conidia per site. A conidium was scored as germinated if the germ tube had reached at least half the length of the conidium. Three plates for each concentration were used and the experiment was performed thrice, along with 98% chlorothalonil (Syngenta Crop Protection Co., Ltd., China) as a positive control. The EC_50_ for inhibition of spore germination was calculated for each isolate. Analysis of parameters was made with the statistical analysis system (SAS institute, Inc., Cary, NC, USA) [21].

## 4. Conclusions

In summary, nine new carabrone derivatives were synthesized and evaluated *in vitro* against *Colletotrichum lagenarium* Ell *et* Halst. Compounds **6**-**8**, and **12 **displayed the more potent antifungal activity than **1**. Meanwhile, the structure-activity relationship (SAR) demonstrated that a *γ*-lactone moiety was necessary for the antifungal activity of **1**, and the substituents on the C-4 position of **1 **could significantly affect their antifungal activity, e.g*.*, introduction of the hydrazone substituents on the C-4 position of **1 **lead to more potent compounds. 
